# A Covalently Micro-Crosslinked Anionic Copolymer-Based Microgel for High-Temperature and Salt-Tolerant Water-Based Drilling Fluids

**DOI:** 10.3390/gels12070588

**Published:** 2026-07-02

**Authors:** Haokun Shen, Jinsheng Sun, Zhenhua Zhang, Xin Zhang, Weijun Yan, Rugang Yao, Hongyan Du, Yuan Geng, Guowei Zhou, Yihua Xu, Yang Zhang

**Affiliations:** 1 CNPC Great Wall Drilling Company, Beijing 100101, China; 2 Key Laboratory of Unconventional Oil & Gas, Development Ministry of Education, Qingdao 266580, China; 3 CNPC Engineering Technology R&D Company Ltd., Beijing 102206, China

**Keywords:** fluid-loss additive, water-based drilling fluid, microgels, high-temperature stability, salt resistance

## Abstract

Fluid-loss additives play a critical role in maintaining the stability and filtration-control performance of water-based drilling fluids during deep and high-temperature drilling operations. However, the development of microgel-based additives with both exceptional thermal stability and strong salt tolerance remains a major challenge under harsh drilling conditions. In this study, a covalently micro-crosslinked anionic copolymer-based microgel (PAAN) was synthesized via free-radical copolymerization of acrylamide, 2-acrylamido-2-methylpropane sulfonic acid, and N-vinylpyrrolidone using N,N′-methylenebisacrylamide as the crosslinking agent. The chemical structure of PAAN is consistent with the design, with excellent thermal stability, and the starting temperature for thermal decomposition of polymer molecular chains is 297 °C. The weight average molecular weight of PAAN is 1.3396 × 10^6^ g/mol. After aging at 220 °C in the presence of 15 wt% NaCl, the PAAN-containing drilling fluid exhibited a high-temperature high-pressure filtration loss of only 15.6 mL. Even after prolonged aging for 168 h, the filtration loss remained at a relatively low level of 46.0 mL, indicating outstanding thermal stability and salt tolerance. Mechanistic analysis indicated that PAAN adsorbed onto bentonite surfaces through electrostatic interactions and hydrogen bonding, promoting clay-particle dispersion and colloidal stability. Moreover, the microgel network facilitated the formation of a compact and low-permeability filter cake, contributing to effective fluid-loss control under harsh conditions. These results demonstrate that microgel structural design is an effective strategy for improving the high-temperature and salt-resistant filtration-control performance of WBDFs, and PAAN shows strong potential for deep and ultra-deep drilling applications.

## 1. Introduction

With the continuous expansion of oil and gas exploration into deep and ultra-deep formations, drilling operations are increasingly conducted under harsh conditions characterized by high temperature and high salinity [1,2]. These extreme environments pose significant challenges to the stability and performance of drilling-fluid systems. Water-based drilling fluids (WBDFs) are widely used in drilling operations because of their low cost, environmental compatibility, and operational flexibility [1,3]. However, under high-temperature and high-salinity conditions, the rheological properties and filtration-control performance of WBDFs often deteriorate markedly, which limits their application in deep and ultra-deep drilling environments.

Fluid-loss additives are essential components of WBDFs because they reduce the invasion of drilling fluids into formations by adsorbing onto bentonite particles and promoting the formation of dense, low-permeability filter cakes [4,5,6]. The effectiveness of these additives largely depends on the polymer molecular structure, the stability of polymer-chain conformations, and intermolecular interactions in highly electrolytic environments. Therefore, the development of polymer-based fluid-loss additives with both high thermal stability and strong salt tolerance remains a major challenge.

Polymer fluid-loss additives can generally be classified into natural polymer derivatives and synthetic polymers. Natural polymers such as starch and cellulose are attractive because of their abundance and environmental friendliness [7,8,9]. However, the glycosidic bonds in their molecular backbones are susceptible to hydrolysis and thermal degradation at elevated temperatures, resulting in limited thermal stability. Although chemical modifications such as esterification, etherification, and graft copolymerization can partially improve their performance, their intrinsic structural limitations still hinder long-term stability under high-temperature conditions [10,11,12,13,14].

Compared with natural polymers, synthetic polymer additives offer greater flexibility in molecular design. By incorporating temperature-resistant monomers, hydrophilic functional groups, and rigid cyclic structures, the thermal stability of polymers can be significantly improved [15,16,17,18]. In recent years, the temperature tolerance of synthetic polymer-based fluid-loss additives has been extended to approximately 200 °C. However, under high-salinity conditions, polymer chains often undergo conformational shrinkage because of electrolyte shielding and reduced intermolecular repulsion, which weakens their dispersion stability and adsorption capacity on bentonite particles [19]. As a result, filtration-control performance deteriorates. Achieving both high thermal resistance and strong salt tolerance in polymer-based fluid-loss additives therefore remains a key challenge.

To address this issue, several strategies have been proposed through regulation of polymer molecular structures and intermolecular interactions. Introducing strongly hydrophilic anionic groups, such as sulfonic acid groups, can enhance polymer solubility and electrolyte tolerance in saline environments [20,21]. Meanwhile, crosslinked structures can effectively restrict excessive chain mobility at elevated temperatures, thereby improving thermal stability [22,23]. Among these strategies, covalent micro-crosslinking has attracted increasing attention because it can enhance polymer structural stability without significantly compromising water solubility [24]. Unlike highly crosslinked three-dimensional gel networks, covalently micro-crosslinked polymers are formed by precisely controlling the crosslinking density, thereby avoiding macroscopic gelation. The localized crosslinking points act as structural constraint nodes along the polymer chains, suppressing conformational relaxation and chain collapse under high-temperature and high-salinity conditions [25]. When combined with anionic functional groups, such micro-crosslinked polymers are expected to exhibit improved thermal stability, salt tolerance, and dispersion stability while maintaining strong interactions with charged clay particles [26,27]. These characteristics provide a promising structural basis for the development of high-performance fluid-loss additives for WBDF systems.

Based on these considerations, a covalently micro-crosslinked anionic copolymer was designed and synthesized in this study and evaluated as a fluid-loss additive for WBDFs. The polymer was constructed by incorporating thermally stable amide groups, rigid cyclic units, and hydrophilic sulfonic groups into the polymer backbone, while a multifunctional crosslinking agent was introduced to generate a low-density covalently micro-crosslinked network. The rheological properties and filtration-control performance of WBDFs containing this copolymer were systematically investigated under coupled high-temperature and high-salinity conditions. Furthermore, the fluid-loss control mechanism was analyzed through microstructural observations and zeta-potential measurements. This work provides new insights into the molecular design of high-temperature- and salt-resistant fluid-loss additives for WBDF systems.

## 2. Results and Discussion

### 2.1. Optimization of MBA Content for PAAN Preparation

The MBA content is a key factor governing the crosslinking density of PAAN microgels, which can significantly influence their structural stability, hydration/swelling behavior, particle deformability, and filtration-control performance. An appropriate crosslinking density is required to maintain the integrity of the microgel network under high-temperature and high-salinity conditions while preserving sufficient flexibility for adsorption and pore plugging. Therefore, the effect of MBA content on the performance of PAAN was first investigated to determine the optimal crosslinking density. A series of PAAN samples with different MBA contents were synthesized by keeping the amounts of AMPS, AM, NVP, KPS, solvent, reaction temperature, and reaction time constant, while only changing the amount of MBA.

As shown in Table 1, the MBA dosage had a pronounced effect on the filtration-control performance of PAAN-containing drilling fluids. With increasing MBA dosage, the high-temperature high-pressure filtration loss (FL_HTHP_) first decreased and then increased. The drilling fluid containing PAAN-0 showed relatively high filtration loss, indicating that the non-crosslinked polymer structure was insufficient to maintain stable filtration-control performance under harsh conditions. When the MBA dosage increased to 0.025 g, the filtration loss decreased, which can be attributed to the formation of a partially crosslinked microgel network with improved structural stability. The lowest filtration loss was obtained for PAAN-0.050, with a FL_HTHP_ of 23.4 mL. Meanwhile, the PAAN-0.050-containing drilling fluid maintained suitable rheological properties, with AV, PV, and YP values of 49.0 mPa·s, 36.0 mPa·s and 13.0 Pa, suggesting that this MBA dosage provided the most favorable balance between filtration control and rheological regulation.

However, further increasing the MBA dosage to 0.075 and 0.100 g did not further improve the filtration-control performance. Instead, the FL_HTHP_ increased to 29.6 and 38.6 mL, respectively. This phenomenon may be ascribed to excessive crosslinking, which restricts the hydration and swelling capacity of the microgel particles and reduces their flexibility and deformability. Highly crosslinked microgel particles are less able to adsorb effectively onto bentonite surfaces or deform to fill micro- and nano-scale pores within the filter cake, resulting in a less compact filtration barrier.

These results indicate that the optimal MBA content does not correspond to the highest crosslinking density, but rather to a balanced microgel architecture that combines thermal/salt stability with sufficient hydration and deformability. Among the investigated samples, PAAN-0.050 exhibited the best overall performance and was therefore selected as the optimized PAAN microgel for subsequent structural characterization, drilling-fluid performance evaluation, and mechanism analysis unless otherwise specified.

### 2.2. Structural Characterization of PAAN

**Figure 1:**
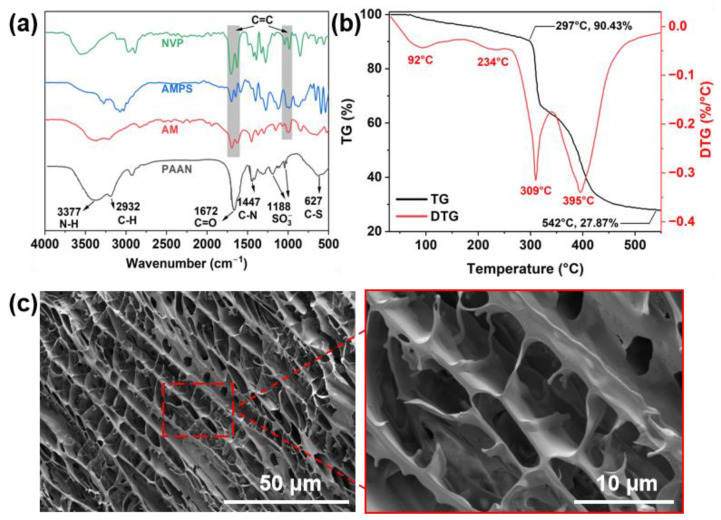
Characterization of PAAN. (**a**) FTIR spectrum confirming the formation of the copolymer structure. (**b**) TG–DTG curves showing the thermal stability of PAAN. (**c**) Cryo-SEM images showing the three-dimensional interconnected porous network of PAAN.

As illustrated in Figure 1a, the FTIR spectrum of PAAN shows a broad band near 3377 cm^−1^, which can be assigned to the stretching vibration of N–H bonds in amide groups. The absorption signal observed at 1672 cm^−1^ is associated with C=O stretching from amide and lactam structures, suggesting the successful introduction of AM, AMPS, and NVP segments into the copolymer chain. Moreover, the bands appearing at 1188 and 1040 cm^−1^ correspond to the asymmetric and symmetric stretching vibrations of –SO_3_^−^ groups, respectively. The peak at 627 cm^−1^ is related to C–S bond vibration, further supporting the incorporation of AMPS units into the PAAN structure. The disappearance of the vinyl C=C signal further confirms the effective conversion of the monomers during polymerization and the successful formation of the PAAN copolymer.

The thermal stability of PAAN was further evaluated, and the results are presented in Figure 1b. A slight weight loss occurs below 120 °C, which is mainly attributed to the evaporation of physically adsorbed and bound water. At 297 °C, the sample still retains 90.43% of its initial mass, suggesting that the copolymer possesses good thermal stability in the moderate temperature range. As the temperature increases further, a noticeable weight-loss stage appears at approximately 300–400 °C, which is mainly associated with the thermal decomposition of amide groups and sulfonic side chains, whereas further degradation of the polymer backbone occurs at around 395 °C. In addition, a residual mass of 27.87% remains at 542 °C, which may be attributed to the formation of relatively stable carbonaceous residues during high-temperature pyrolysis. It should be noted that the target application temperature of PAAN in this study is 220 °C, which is lower than the main decomposition temperature observed in the TGA curve. In many studies on high-temperature drilling-fluid additives, TGA is used to evaluate the intrinsic thermal degradation behavior of dried polymers under programmed heating conditions, and the decomposition temperature is commonly compared with a lower service or thermal-aging temperature [28]. Therefore, the fact that PAAN shows no obvious main decomposition below 220 °C suggests that the copolymer possesses sufficient intrinsic thermal resistance for the intended high-temperature drilling-fluid application.

GPC analysis was performed to evaluate the molecular weight. As shown in Figure S1, PAAN exhibited an apparent number-average molecular weight (Mn) of 7.1618 × 10^5^ g/mol and an apparent weight-average molecular weight (Mw) of 1.3343 × 10^6^ g/mol. The relatively high apparent molecular weight is beneficial for improving the viscosity-building ability of PAAN in water-based drilling fluids.

The particle size distribution of the optimized PAAN was measured by DLS. As shown in Figure S2, PAAN exhibits a continuous particle size distribution mainly ranging from approximately 200 to 800 nm, with a dominant peak at around 390 nm. The measured particle size represents the hydrodynamic diameter of PAAN in the swollen state, confirming the formation of hydrated microgel particles in aqueous dispersion. This particle size distribution is beneficial for adsorption onto bentonite surfaces and for filling pores within the filter cake, thereby promoting the formation of a compact, low-permeability filtration barrier and improving the filtration-control performance of WBDFs.

To further investigate the microstructural characteristics of PAAN, cryo-scanning electron microscopy (Cryo-SEM) was employed. As shown in Figure 1c, PAAN exhibits a continuous three-dimensional porous network with interconnected pores and relatively uniform pore walls. These morphological features indicate that the polymer possesses distinct network characteristics. Because a multifunctional crosslinking agent was introduced during polymerization, this structure may be associated with the formation of a covalently micro-crosslinked network with a low crosslink density. The localized crosslinking points may partially restrict excessive polymer-chain mobility while maintaining the overall structural integrity of the network.

### 2.3. Performance Evaluation of PAAN in Drilling Fluids

**Figure 2:**
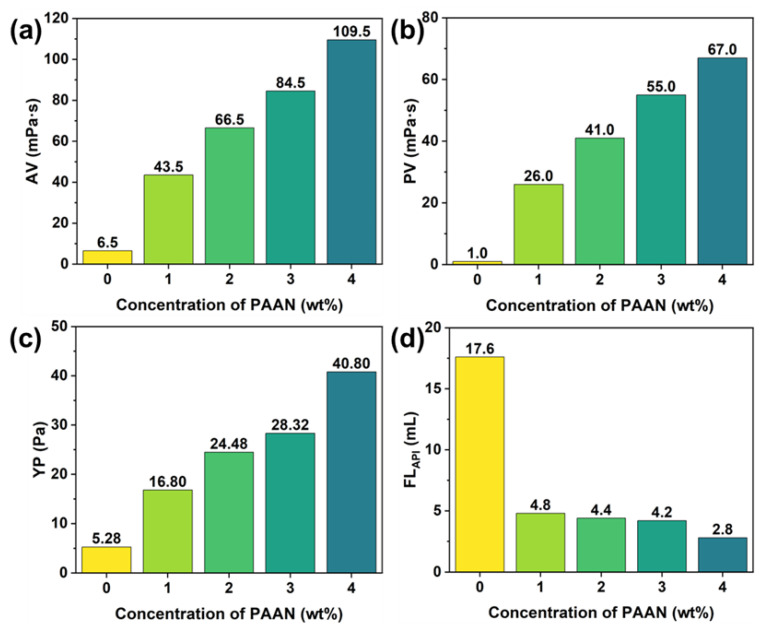
Effect of PAAN concentration on the rheological and filtration properties of bentonite-based drilling fluid: (**a**) apparent viscosity; (**b**) plastic viscosity; (**c**) yield point; (**d**) API filtration loss.

The influence of PAAN concentration on the rheological properties and filtration performance of a 4 wt% bentonite-based drilling fluid is presented in Figure 2. As the PAAN concentration increases from 0 to 4 wt%, the rheological parameters of the drilling fluid, including apparent viscosity (AV), plastic viscosity (PV), and yield point (YP), increase markedly.

As shown in Figure 2a–c, the increase in AV reflects higher flow resistance under shear, which is beneficial for improving the cuttings-carrying capacity of the bentonite-based drilling fluid. Meanwhile, the increase in PV suggests stronger interactions between PAAN molecules and bentonite particles, leading to the formation of a more stable polymer–clay network. The pronounced increase in YP further indicates improved structural integrity of the drilling fluid, which helps maintain cuttings suspension and prevents particle settling under low-shear or static conditions. The corresponding filtration performance is shown in Figure 2d. With increasing PAAN concentration, FL_API_ decreased markedly from 17.6 to 2.8 mL, while the API filtration-loss reduction increased from 72.7% to 84.1% relative to the blank formulation (Figure S3a), confirming the concentration-dependent filtration-control capability of PAAN. This improvement can be attributed to the adsorption of PAAN onto bentonite particle surfaces, which enhances interparticle interactions and facilitates the formation of a denser, less permeable filter cake. These results indicate that PAAN effectively improves both the rheological properties and the filtration-control performance of the bentonite-based drilling fluid.

**Figure 3:**
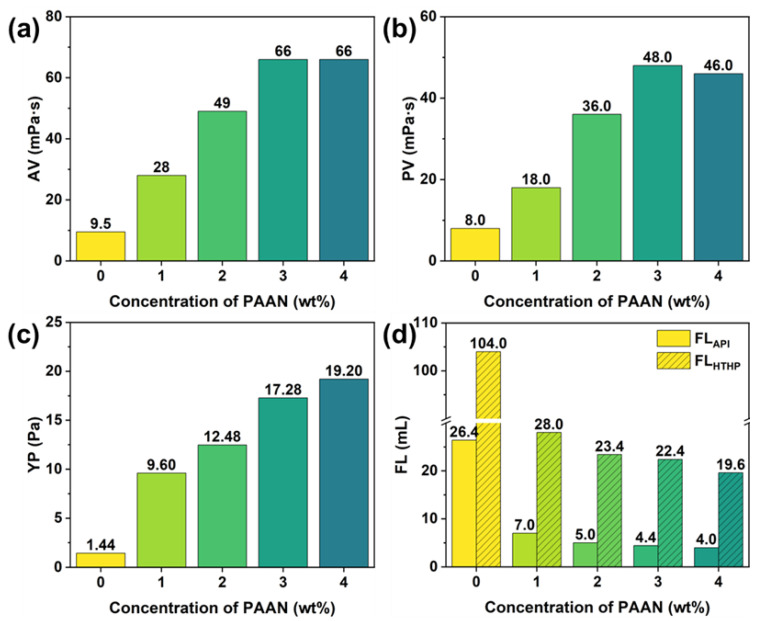
Effect of PAAN concentration on the rheological and filtration properties of bentonite-based drilling fluid after aging at 220 °C for 16 h: (**a**) AV; (**b**) PV; (**c**) YP; (**d**) Filtration performance.

The rheological response and filtration-control behavior of bentonite-based drilling fluids after thermal aging at 220 °C for 16 h are shown in Figure 3. As the PAAN dosage increased, the AV, PV, and YP values exhibited a continuous upward trend, demonstrating that PAAN was able to reinforce the internal network of the drilling-fluid system even after severe heat treatment. However, the rise in PV became gradually less evident at relatively high polymer dosages, implying that the interaction between PAAN and clay particles tended toward a stable equilibrium, as illustrated in Figure 3a–c.

As shown in Figure 3d, both FL_API_ and FL_HTHP_ decrease significantly with increasing PAAN concentration. At a PAAN concentration of 4 wt%, FL_HTHP_ decreased from 104.0 to 19.6 mL, corresponding to a reduction rate of 81.2% relative to the blank formulation (Figure S3b). Meanwhile, the reduction FL_API_ increased from 73.5% to 84.8% as the PAAN concentration increased from 1 to 4 wt% (Figure S3b). These results indicate that PAAN can effectively maintain structural stability and adsorption capacity after high-temperature aging, thereby promoting the formation of a dense, low-permeability filter cake and preserving the overall performance of the drilling-fluid system [29,30].

### 2.4. Effect of Salinity on the Performance of PAAN-Based Drilling Fluids

In practical drilling operations, water-based drilling fluids are often exposed to saline formations or salt contamination, which can significantly influence bentonite hydration, polymer conformation, and polymer–clay interactions. These changes may further affect the rheological properties and filtration performance of the drilling fluid. Therefore, evaluating the effect of NaCl concentration is essential for assessing the salt tolerance and practical application potential of the PAAN-based drilling-fluid system.

**Figure 4:**
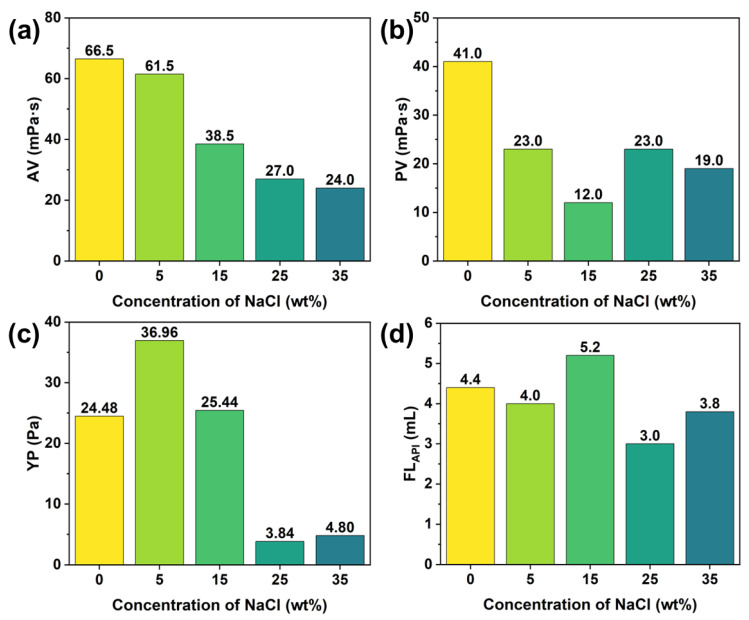
Effects of NaCl concentration on the rheological properties and filtration performance of PAAN-based drilling fluid: (**a**) AV; (**b**) PV; (**c**) YP; (**d**) API filtration loss. The concentration of PAAN is fixed at 2 wt%.

As the NaCl concentration increases, the AV of the PAAN-based drilling fluid generally decreases (Figure 4a). This behavior is mainly attributed to the enhanced electrolyte-shielding effect at higher salinity, which weakens the electrostatic interactions between polymer chains and bentonite particle surfaces. Meanwhile, partial contraction of the polymer chains in the saline environment may further reduce the overall viscosity of the system. PV initially decreases at low salinity and then increases slightly at higher NaCl concentrations (Figure 4b). This trend suggests that the polymer–clay structure undergoes structural rearrangement rather than complete collapse under saline conditions. At low salinity, the shielding effect reduces electrostatic repulsion between particles, leading to partial loosening of the system structure, whereas at higher salinity, interparticle interactions may gradually reach a new equilibrium state. YP reaches a maximum at moderate salinity, indicating enhanced particle-interaction strength at appropriate electrolyte concentrations. However, with further increases in NaCl concentration, YP gradually decreases, suggesting that excessive salinity weakens the structural strength of the system (Figure 4c).

Notably, as shown in Figure 4d, the FL_API_ remained relatively low over the entire NaCl concentration range. Even under high-salinity conditions, the PAAN-based drilling fluid still exhibited effective filtration control performance. This result indicates that PAAN can still contribute to the formation of a relatively dense filter cake in saline environments, thereby maintaining low filter cake permeability [31].

**Figure 5:**
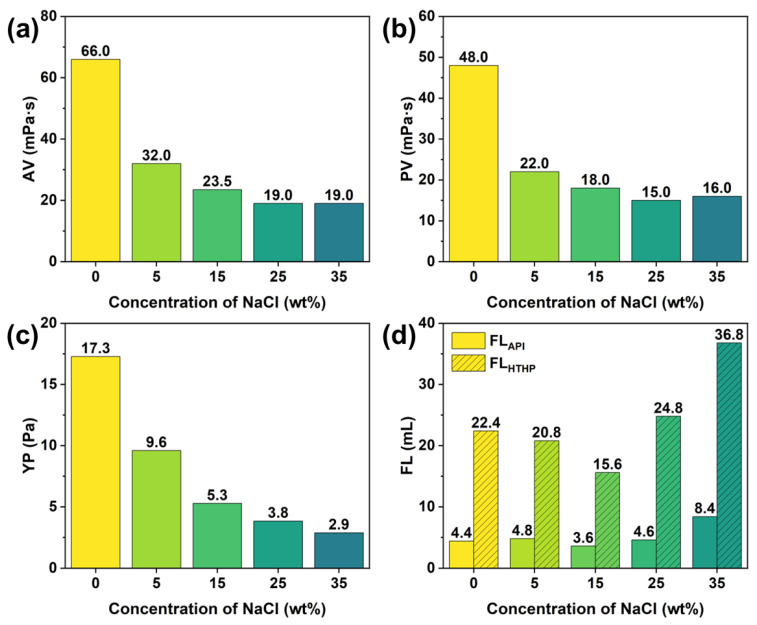
Effects of NaCl concentration on the rheological parameters and filtration loss of PAAN-based drilling fluid after aging at 220 °C: (**a**) AV; (**b**) PV; (**c**) YPt; (**d**) Filtration loss. The concentration of PAAN was fixed at 2 wt%.

As shown in Figure 5, the rheological and filtration properties of PAAN-based drilling fluids were evaluated under coupled high-temperature and salinity stresses. Increasing NaCl concentration generally resulted in lower AV, PV, and YP values. This trend is likely caused by electrolyte shielding together with enhanced thermal movement of polymer chains, both of which weaken the polymer–clay framework and reduce the structural stability of the drilling fluid.

Despite this decrease, the rheological parameters tend to stabilize at higher salinity levels, indicating that the system undergoes structural compression and rearrangement rather than complete structural collapse. In terms of filtration performance, API filtration loss remains relatively low across the salinity range, while FL_HTHP_ increases gradually with increasing NaCl concentration. This suggests that increasing salinity may partially weaken the contribution of the polymer to the filter-cake structure. Nevertheless, the filtration performance remains within an acceptable range even at high salinity.

These results indicate that PAAN can maintain a certain degree of structural stability and still contribute to the formation of a relatively dense filter cake under high-temperature and high-salinity conditions.

### 2.5. Long-Term Thermal Aging Stability

In practical drilling operations, drilling fluids may remain exposed to high-temperature and high-salinity environments for extended periods, during which the polymer structure and polymer–clay interactions may gradually deteriorate. Therefore, long-term aging tests are essential for evaluating the durability and practical applicability of the fluid-loss additive [32].

**Figure 6:**
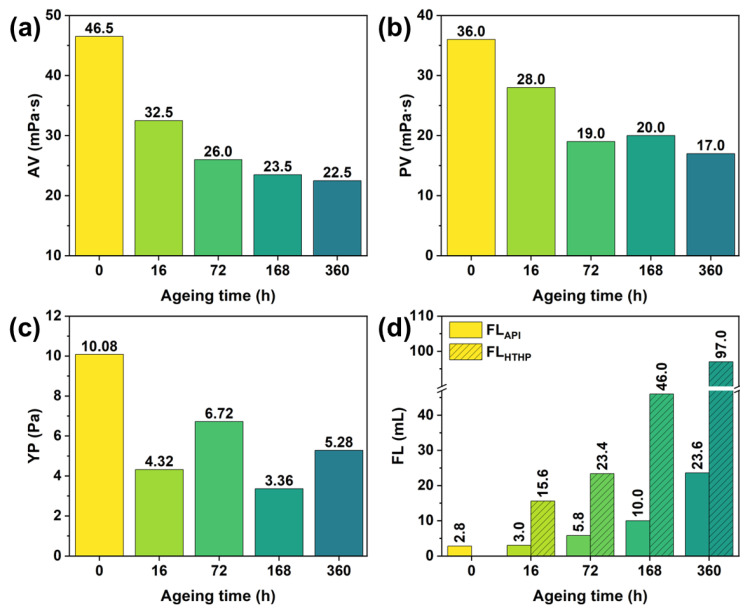
Effects of aging time on the rheological parameters and filtration performance of PAAN-based drilling fluids at 220 °C with 15 wt% NaCl. (**a**) AV. (**b**) PV. (**c**) YP. (**d**) Filtration loss. The concentration of PAAN was fixed at 2 wt%.

With increasing aging duration, the AV, PV, and YP values of the drilling fluid decreased gradually, as illustrated in Figure 6a–c. This trend indicates that extended exposure to severe aging conditions weakened the interactions within the polymer–clay framework to a certain extent. This variation may be associated with molecular-level changes in PAAN during long-term thermal aging. For acrylamide-based polymers, amide groups are susceptible to partial hydrolysis under high-temperature aqueous conditions, leading to the formation of carboxyl/carboxylate groups and changes in anionic charge density [33]. Moderate hydrolysis may enhance electrostatic repulsion and polymer–bentonite interactions, whereas excessive hydrolysis, together with thermal/oxidative chain degradation, may reduce molecular integrity and weaken the network-forming ability of the polymer [34]. However, after extended aging, the rheological parameters tended to stabilize, suggesting that the system reached a new structural equilibrium rather than undergoing abrupt structural failure.

Meanwhile, both the FL_API_ and the FL_HTHP_ increased with increasing aging time, reflecting a gradual decline in filtration control performance (Figure 6d). This increase can be attributed to the progressive weakening of PAAN adsorption, polymer–clay association, and filter-cake plugging efficiency caused by long-term thermal stress. Nevertheless, the variation in filtration behavior remained relatively moderate and predictable. Even after long-term aging at 220 °C, the PAAN-containing system still maintained effective filtration-control capability, which can be attributed to the synergistic contribution of the thermally stable AMPS and NVP units as well as the covalently micro-crosslinked architecture. The sulfonate groups in AMPS provide strong hydration and salt tolerance, while NVP units contribute to the thermal stability of the copolymer structure. In addition, the micro-crosslinked network helps preserve the structural integrity of PAAN and mitigates complete chain collapse under prolonged thermal stress. Therefore, although partial hydrolysis and thermal degradation may occur during extended aging, PAAN can still retain sufficient adsorption, deformation, and filter-cake densification ability under harsh conditions.

### 2.6. Mechanism Analysis of Filtration Reduction by PAAN

During the filtration of drilling fluids, solid components are progressively trapped on the wellbore surface, resulting in the development of a filter cake. The integrity of this deposited layer is essential for fluid-loss control. Generally, a high-quality filter cake should exhibit a dense and continuous structure with minimal pores and cracks, thereby effectively reducing the invasion of drilling fluid into the formation.

**Figure 7:**
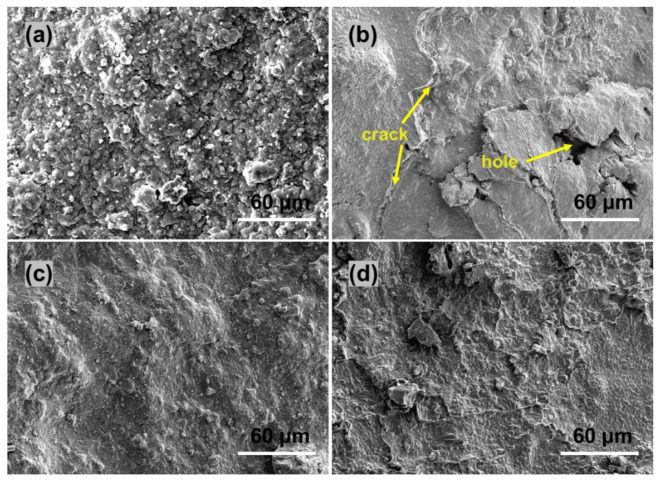
SEM images of filter cakes formed from different drilling fluids. (**a**) Bentonite-based drilling fluid. (**b**) Bentonite-based drilling fluid with 15 wt% NaCl. (**c**) PAAN-based drilling fluid. (**d**) PAAN-based drilling fluids with 15 wt% NaCl.

Figure 7 presents the SEM images of filter cakes formed by different drilling fluid systems after aging. For the bentonite-based drilling fluid without polymer additives, the resulting filter cake structure is relatively loose and porous (Figure 7a). Under NaCl contamination conditions, obvious cracks and pores appear on the surface of the filter cake, indicating that the saline environment weakens the structural integrity of the filter cake and deteriorates its quality (Figure 7b). In contrast, the filter cake formed in the presence of PAAN becomes significantly denser and more uniform, showing a more continuous morphology with substantially fewer structural defects (Figure 7c). Even under NaCl contamination conditions, the drilling fluid system containing PAAN still maintains a relatively compact filter cake structure, with significantly reduced numbers of cracks and pores (Figure 7d). These observations suggest that PAAN enhances the interactions between polymer chains and clay particles, thereby promoting tighter particle packing within the filter cake. As a result, the microstructure of the filter cake is improved, its permeability is reduced, and effective filtration control is achieved.

**Figure 8:**
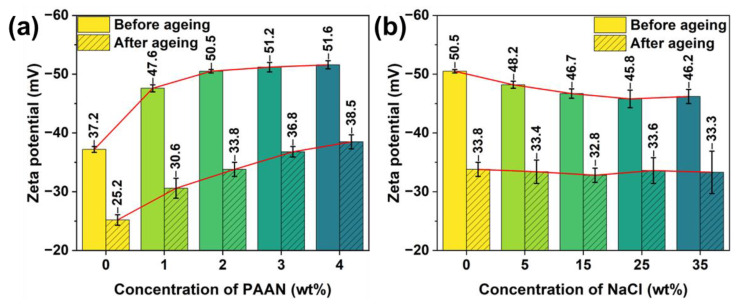
Zeta potential of bentonite-based drilling fluids as a function of PAAN concentration and NaCl concentration before and after aging at 220 °C. (**a**) PAAN concentration. (**b**) NaCl concentration.

Figure 8 shows the effects of PAAN concentration and NaCl concentration on the zeta potential of bentonite-based drilling fluids before and after aging at 220 °C. As the PAAN concentration increases, the absolute value of the zeta potential increases significantly. This behavior may be attributed to the adsorption of anionic functional groups in PAAN onto bentonite particle surfaces, which increases the negative surface-charge density and enhances electrostatic repulsion between particles. Although high-temperature aging slightly reduces the absolute value of the zeta potential, the systems containing PAAN still maintain relatively high negative zeta-potential values, suggesting that the polymer–clay interactions remain relatively stable even under high-temperature conditions [35]. Furthermore, even under high-salinity conditions, the variation in zeta potential with increasing NaCl concentration remains relatively small. This result indicates that PAAN can partially mitigate the electrolyte-shielding effect and help maintain the dispersion stability of clay particles in saline environments. Consequently, the stable dispersion of clay particles contributes to the formation of a dense filter cake, thereby improving filtration-control performance [36].

**Figure 9:**
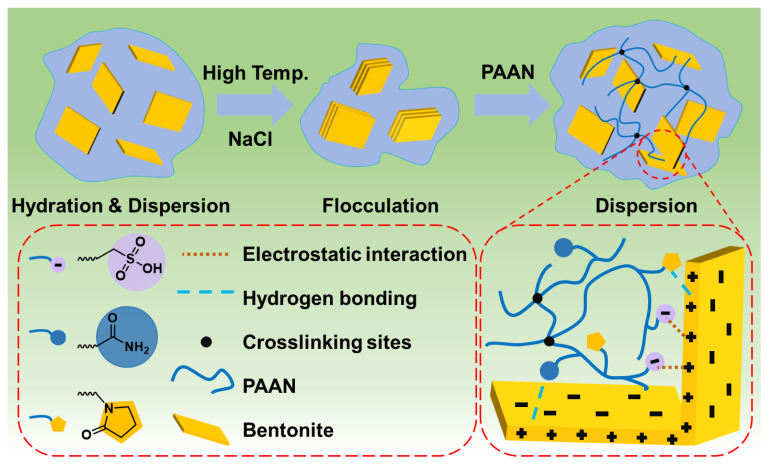
Schematic illustration of the fluid-loss control mechanism of PAAN in bentonite-based drilling fluids.

Based on the above experimental results, a schematic illustration of the fluid-loss control mechanism of PAAN is proposed (Figure 9). The filtration-control performance of PAAN is attributed to the synergistic effects of electrostatic interactions, hydrogen bonding, and the covalently micro-crosslinked polymer network. The anionic functional groups in PAAN can adsorb onto the surfaces of bentonite particles, thereby enhancing electrostatic repulsion between particles and improving dispersion stability. Meanwhile, hydrogen bonding further stabilizes the interactions between polymer chains and clay particles, especially under high-temperature conditions. In addition, the covalently micro-crosslinked polymer network helps restrict excessive contraction of polymer chains under high-temperature and high-salinity conditions, thereby maintaining the conformational stability of the polymer molecules. Through these synergistic effects, PAAN promotes the formation of a dense, low-permeability filter cake and maintains effective filtration-control performance even under harsh drilling conditions.

## 3. Conclusions

In this study, a covalently micro-crosslinked anionic copolymer-based microgel (PAAN) was successfully synthesized and systematically investigated for application in WBDFs. Structural characterization confirmed the successful incorporation of amide and sulfonic functional groups into the polymer backbone and verified the formation of a covalently micro-crosslinked microgel architecture. This microgel network endowed PAAN with enhanced structural integrity and resistance to thermal degradation and salt-induced destabilization, providing the basis for its excellent performance under harsh conditions. Performance evaluations demonstrated that PAAN significantly improved the rheological properties and filtration-control capability of WBDFs. Even under severe conditions involving high temperature, high salinity, and prolonged aging, the PAAN-containing drilling fluids maintained favorable rheological stability and effective fluid-loss control, highlighting the adaptability of the microgel system to demanding drilling environments. Mechanistic investigations revealed that the superior fluid-loss control performance of PAAN originated from the synergistic effects of electrostatic interactions, hydrogen bonding, and the covalently micro-crosslinked microgel network. The microgel architecture enhanced the dispersion stability of bentonite particles, strengthened colloidal stability, and facilitated the formation of a compact and low-permeability filter cake, thereby effectively reducing filtration loss. Overall, the present work demonstrates that covalently micro-crosslinked microgels represent a promising class of functional gel materials for drilling-fluid applications under extreme conditions. Beyond their practical potential in deep and ultra-deep drilling operations, these findings provide new insights into the structure–function relationships of microgel systems and offer useful guidance for the molecular design of high-performance functional microgels with enhanced thermal and salt resistance.

## 4. Materials and Methods

### 4.1. Materials

Acrylamide (AM), 2-acrylamido-2-methylpropane sulfonic acid (AMPS), N-vinylpyrrolidone (NVP), N,N′-methylenebisacrylamide (MBA), and anhydrous ethanol were purchased from Macklin Reagent Co., Ltd. (Shanghai, China). Sodium chloride (NaCl), sodium hydroxide (NaOH), potassium persulfate (KPS), and anhydrous sodium carbonate (Na_2_CO_3_) were obtained from Aladdin Reagent Co., Ltd. (Shanghai, China). Bentonite was supplied by Bohai Drilling Engineering Co., Ltd. (CNPC, Tianjin, China). Deionized water was prepared in the laboratory and used throughout all experiments. Unless otherwise specified, all chemicals were of analytical grade and used as received.

### 4.2. Preparation of Drilling Fluids

#### 4.2.1. Bentonite-Based Drilling Fluid

The bentonite-based drilling fluid was prepared by gradually adding 16.0 g bentonite into 400 mL deionized water under high-speed stirring. Subsequently, 0.48 g Na_2_CO_3_ was added as a dispersing agent. The suspension was continuously stirred for 20 min and then aged for 24 h at room temperature to allow full hydration of bentonite.

#### 4.2.2. PAAN-Based Drilling Fluid

A predetermined amount of PAAN powder was added into the pre-hydrated bentonite-based drilling fluid under high-speed stirring. The mixture was stirred for 20 min and then aged for 24 h to obtain a homogeneous polymer-based drilling fluid.

### 4.3. Synthesis of the Fluid-Loss Additive PAAN

**Figure 10:**
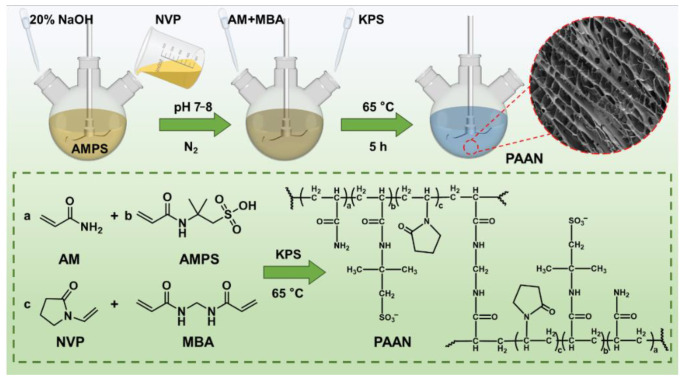
Schematic illustration of the synthesis and formation mechanism of the covalently micro-crosslinked anionic copolymer PAAN.

The covalently micro-crosslinked anionic copolymer (PAAN) was synthesized via free-radical copolymerization. First, 100 g deionized water and 12.2 g AMPS were added into a three-neck round-bottom flask equipped with a mechanical stirrer, thermometer, and nitrogen inlet. After AMPS was completely dissolved, the pH of the solution was adjusted to 7–8 using a 20 wt% NaOH solution. Subsequently, 3.6 g NVP was added to the reaction system and the temperature was increased to 65 °C. Subsequently, 3.6 g NVP was added to the reaction system, and the temperature was increased to 65 °C under a nitrogen atmosphere. Then, a monomer/crosslinker solution containing 8.6 g AM and 0.05 g MBA dissolved in 50 g deionized water and an initiator solution containing 0.006 g KPS dissolved in 10 mL deionized water were added concurrently into the reactor over 1 h using separate dropping funnels. After the simultaneous dropwise addition was completed, the reaction mixture was maintained at 65 °C for 5 h under continuous stirring. After the addition was completed, the reaction mixture was maintained at 65 °C for 5 h under continuous stirring. The entire polymerization process was conducted under a nitrogen atmosphere to remove dissolved oxygen and prevent radical inhibition. After the reaction, the obtained polymer solution was poured into a large amount of anhydrous ethanol to precipitate the polymer and remove unreacted monomers and oligomers. The precipitated polymer was collected by filtration, dried under vacuum at 65 °C, and then ground into powder to obtain the covalently micro-crosslinked anionic copolymer (PAAN). To optimize the crosslinking density of PAAN, a series of PAAN samples were prepared by varying the MBA content while keeping all other synthesis conditions unchanged. Specifically, the MBA contents were set as 0, 0.025, 0.050, 0.075, and 0.100 g, and the corresponding products were denoted as PAAN–0, PAAN–0.025, PAAN–0.050, PAAN–0.075, and PAAN–0.100, respectively. The synthesis route of PAAN is illustrated in Figure 10.

### 4.4. Characterization Methods

The chemical structure of PAAN was characterized by Fourier transform infrared spectroscopy using an IRTracer-100 spectrometer from Shimadzu, Nakagyo-ku, Kyoto, Japan. The samples were prepared using the KBr pellet method, and spectra were recorded in the wavenumber range of 400–4000 cm^−1^ with a resolution of 0.25 cm^−1^.

The thermal stability of PAAN was evaluated using a thermogravimetric analyzer (TGA) (Mettler-Toledo, Greifensee, Switzerland). Approximately 5 mg of sample was placed in a crucible and heated from 30 °C to 550 °C at a heating rate of 10 °C·min^−1^ under a nitrogen atmosphere.

The apparent molecular weight and molecular-weight distribution were measured by an Agilent-1260 Infinity II (Agilent, Santa Clara, CA, USA) equipped with a refractive index detector. 0.2 mol/L NaNO_3_ aqueous solution was used as the mobile phase at a flow rate of 1.0 mL/min. The column temperature was maintained at 35 °C, and the injection volume was 5 mL. Before analysis, the PAAN solution was filtered through a 0.45 μm aqueous membrane.

The particle size distribution of the optimized PAAN microgel was determined by dynamic light scattering (DLS) using a Zetasizer Nano ZS (Malvern Panalytical, Malvern, UK). PAAN was dispersed in deionized water at a concentration of 0.1 g/L and stirred to obtain a homogeneous dispersion before measurement. The test was conducted at 25 °C with a detection angle of 90°. The hydrodynamic particle size distribution was recorded based on the intensity of scattered light.

The microstructure of PAAN was observed using cryo-scanning electron microscopy (Cryo-SEM) (Regulus 8100, Hitachi, Marunouchi, Tokyo, Japan). The cryo-stage temperature was maintained at −140 °C, and the accelerating voltage was set to 5 kV.

### 4.5. Evaluation of Drilling Fluid Performance

All drilling fluid tests were conducted according to API recommended practices.

The rheological properties of drilling fluids were measured using a ZNN-D6 rotational viscometer (Qingdao Tongchun Petroleum Instrument Co., Ltd., Qingdao, China) at 50 °C. The apparent viscosity (AV), plastic viscosity (PV), and yield point (YP) were calculated using the following equations:

(1) $$\mathrm{AV}=0.5\times \theta_{600}\;(\mathrm{mPa}\cdot s)$$

(2) $$\mathrm{PV}=\theta_{600}-\theta_{300}\;(\mathrm{mPa}\cdot s)$$

(3) YP=AV−PV (Pa)

where *θ*_600_ and *θ*_300_ are the viscometer readings at 600 rpm and 300 rpm, respectively.

To evaluate the performance of drilling fluids under thermally harsh conditions, the prepared fluid samples were first subjected to aging treatment in a roller oven at 220 °C for 16 h. After aging, their rheological behavior and filtration properties were measured.

The FL_API_ was determined using an SD6A filter press supplied by Qingdao Tongchun Petroleum Instrument Co., Ltd., China. In each test, 250 mL of drilling fluid was placed in the filtration cell, and standard API filter paper with a pore size of 3 μm was used as the filtration medium. The filtration experiment was performed at 0.75 MPa for 30 min.

The FL_HTHP_ was measured with a high-temperature filter press at 220 °C and 3.50 MPa for 30 min. In addition, to assess the long-term thermal stability of the drilling fluid systems, samples were aged at 220 °C for 16, 72, 168, and 360 h, respectively. Their rheological parameters and filtration-control performance were then evaluated after each aging period.

### 4.6. Mechanism Analysis

Before zeta potential analysis, the drilling fluid samples were diluted 100-fold with deionized water. The zeta potential of the resulting suspensions was determined using a Zetasizer Nano ZS90 instrument (Malvern, UK). For each formulation, three parallel measurements were carried out, and the final result was expressed as the mean value.

Following the API filtration experiment, the collected filter cakes were vacuum-dried at 60 °C until a constant mass was reached. Their surface microstructures were then examined by scanning electron microscopy (EVO 15/LS, ZEISS, Oberkochen, Germany). Prior to SEM characterization, a thin gold layer was sputtered onto the sample surfaces to enhance electrical conductivity. SEM observations were performed at an accelerating voltage of 20 kV.

## Figures and Tables

**Table 1:** Rheological and filtration properties of WBDFs containing PAAN synthesized with different MBA dosages. All PAAN-containing WBDFs were prepared at a fixed concentration of 2 wt% and aged at 220 °C for 16 h.

Sample	AV/mPa·s	PV/mPa·s	YP/Pa	FL_API_/mL	FL_HTHP_/mL
Blank Bentonite-based drilling fluid	9.5	8.0	1.5	26.4	104.0
PAAN-0	36.0	28.0	8.0	9.8	46.6
PAAN-0.025	43.0	33.0	10.0	6.6	31.8
PAAN-0.050	49.0	36.0	13.0	5.0	23.4
PAAN-0.075	55.0	40.0	15.0	5.8	29.6
PAAN-0.100	60.0	42.0	18.0	7.2	38.6

## Data Availability

The original contributions presented in this study are included in the article. Further inquiries can be directed to the corresponding author.

## Supplementary Materials

The following supporting information can be downloaded at: https://www.mdpi.com/article/10.3390/gels12070588/s1, Figure S1: GPC chromatogram and molecular-weight parameters of the PAAN–0.05; Figure S2: Particle size distribution of PAAN–0.05 in deionized water; Figure S3: Percentage reduction in filtration loss of PAAN-containing WBDFs relative to the blank formulation as a function of PAAN concentration: (a) API filtration-loss reduction before aging; (b) API and HTHP filtration-loss reductions after aging at 220 °C for 16 h.
