# Supplementary material for: A Covalently Micro-Crosslinked Anionic Copolymer-Based Microgel for High-Temperature and Salt-Tolerant Water-Based Drilling Fluids
Source: Gels. 2026 Jul 2;12(7):588. doi: 10.3390/gels12070588 (PMC13409077; doi:10.3390/gels12070588)
Supplement: Supplementary file 1 [file gels-12-00588-s001.zip › gels-4401424-supplementary.pdf]

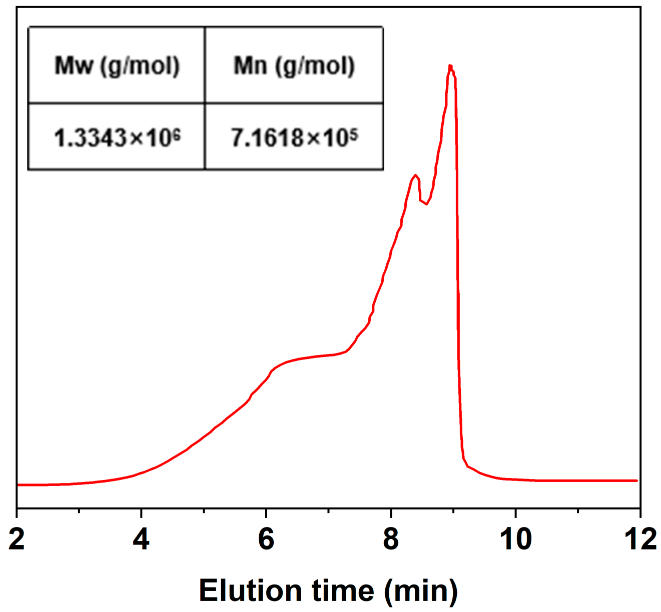

Figure S1. GPC chromatogram and molecular-weight parameters of the PAAN-0.05.

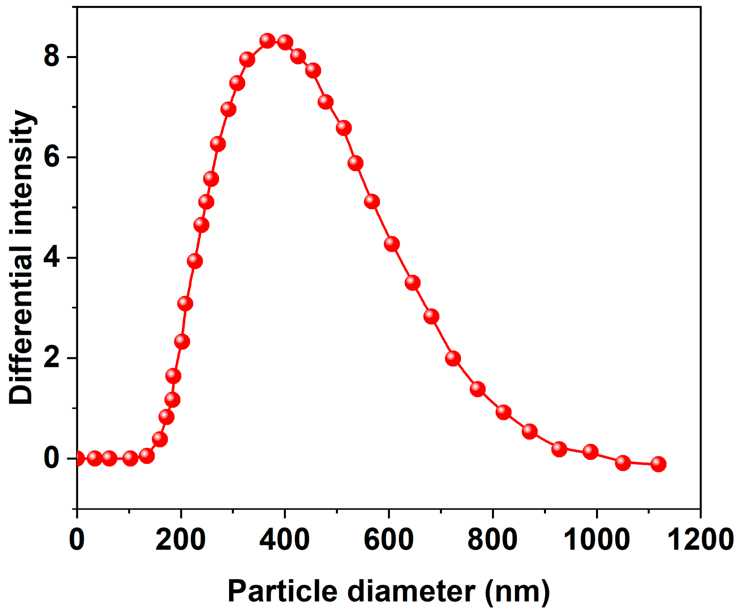

Figure S2. Particle size distribution of PAAN-0.05 in deionized water.

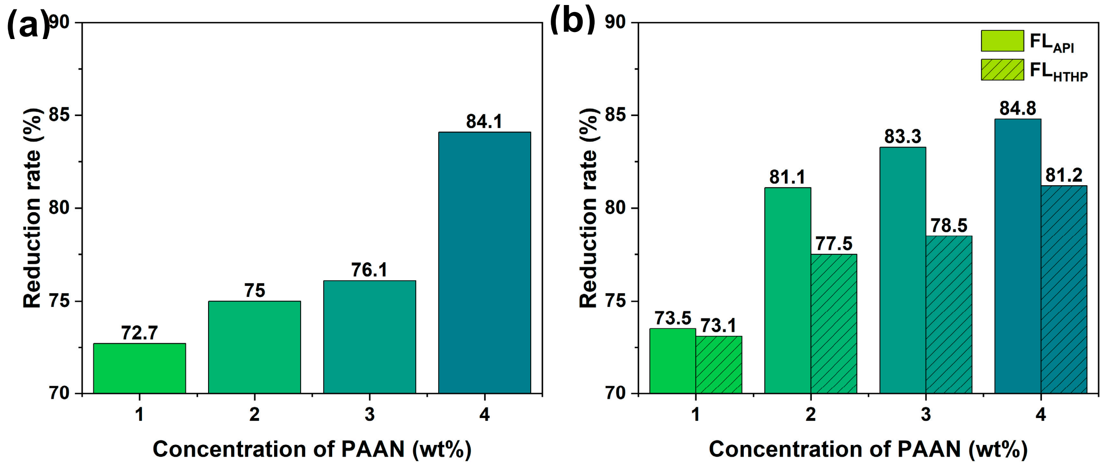

Figure S3. Percentage reduction in filtration loss of PAAN-containing WBDFs relative to the blank formulation as a function of PAAN concentration: (a) API filtration-loss reduction before aging; (b) API and HTHP filtration-loss reductions after aging at 220 °C for 16 h
